# Medical student assessments—frequency of radiological images used: a national study

**DOI:** 10.1259/bjro.20200047

**Published:** 2020-12-11

**Authors:** Cindy Chew, Patrick J O'Dwyer, Alan Jaap, Suanne McDowall, Olga JL Remers, JJZ Williams, I McPhee, Predrag Bjelogrlic

**Affiliations:** 1 Department of Radiology, University Hospital Hairmyres, East Kilbride, UK; 2 School of Medicine, Dentistry and Nursing, University of Glasgow, Glasgow, UK; 3 Edinburgh Medical School, Chancellor’s Building, Edinburgh, UK; 4 School of Medicine, University of Dundee, Ninewells Hospital & Medical School, Dundee, UK; 5 University of Aberdeen School of Medicine and Dentistry, Aberdeen, UK; 6 School of Medicine, University of St Andrews, Medical and Biological Sciences Building, St Andrews, UK

## Abstract

**Objectives:**

Assessments are a key part of life for medical students at University. We know there is variation in these assessments across Universities. The aims of this study were to expatiate summative assessments in Scottish Medical Schools and to examine how frequently radiological images feature in them.

**Methods:**

All Scottish medical schools were invited to participate in the study. Data on objective structured clinical examinations (OSCEs; 5 years) and written assessments (3 years) were retrospectively collected for each university and results were collated. Each University was randomly assigned a letter from A to E and anonymised for data presentation.

**Results:**

10,534 multiple choice questions (MCQ) and 1083 OSCE stations were included in this study. There was wide variation in the number, type and timing of assessments across Scottish medical schools. There were highly significant differences in the number of OSCE stations and the number of MCQs set over the study period (*p* < 0.0001). Radiological images were used on average 0.6 times (range 0–1.1) in each OSCE examination and 2.4 times (range 0.1–3.7) for written assessments.

**Conclusion:**

In this detailed study, we demonstrated significant differences in medical student assessments across Scottish Universities. Given the importance of Radiology in modern medicine, the frequency and differences in which radiological images were used in assessments across Universities should be addressed.

**Advances in knowledge:**

This is the first national longitudinal study to quantify the role of radiological images in summative Medical Student Assessments. Great variability exists in the extent and how (clinical versus written assessments) radiological images are used to assess Scottish medical students. Radiological images are used infrequently in clinical assessments, but are present in every written assessment. These findings could help inform medical schools and academic radiologists as they prepare medical students for the imminent unified medical licensing examination, where Clinical Imaging is a subject with one of the highest number of associated conditions examinable.

## Introduction

Assessments are a key part of life for medical students at University. It is important to ensure tomorrow’s doctors have the core knowledge, skills and behaviours needed to practice safely. The best assessments are comprehensive, mapped clearly to each school’s curriculum and meet standards set out by the General Medical Council (GMC) outcomes for Graduates.^1^ These, as well as how well students are prepared for assessments, are part of how Medical Schools are evaluated by the GMC.^2^ The GMC will deploy the first Medical Licensing Assessment in 2023–24. Medical students will need to pass the MLA as part of their medical degree, before they can join the medical register. While the exact format is yet to be finalised, it will no doubt reflect the myriad of assessment tools currently used by medical schools in the UK.

It is widely acknowledged that complex behavioural skills are required for the medical student to evolve into a competent and safe medical practitioner. This has resulted in the move away from time-based, knowledge-orientated programmes to outcome and competency-based education.

Radiology has long been espoused as an excellent tool for teaching medical students.^3,4^ Not only because Radiology is well placed to teach clinical anatomy, it also plays a vital role in modern medicine and serves therefore as an excellent and important aide to the learning and teaching of clinical reasoning (What is the best test? What does it mean if a test is positive/negative? Do the findings fit in with the clinical picture?). Radiology, however, is infrequently formally included in medical schools’ curricula. There is a gap in the literature regarding the role Radiology plays in medical school assessments. While assessments should not drive learning, a subject’s inclusion highlights its importance.

The aims of this study were to expatiate summative assessments in Scottish Medical Schools and to examine how frequently radiological images feature in them.

## Methods and materials

Institutional board review was sought and waived – this study was considered part of normal course evaluation with no sensitive data being handled.

All five Scottish medical schools were invited to participate in the study in April 2020. The relevant person within each medical school with knowledge of and access to assessment data was contacted. Each representative collected information on written assessments and OSCEs retrospectively for their university and results were collated. Due to the way data were archived in one of the Universities, information was limited to 3 years of written assessments (2017–19) and 5 years of OSCE data (2015–19). Data on what specific radiological images used were not collected because of potential impact on future assessments. However, a wide range of images were used; for example for anatomy, X-rays of bones and joints, CT images of brain, thorax, abdomen and pelvis, MRI of soft tissues, angiographical and cholangiographical images were utilised. Clinical assessments focused on Chest X-rays of common pathologies, common fractures and gross abnormalities visible on CT scans. Each University was randomly assigned a letter from A to E and anonymised for the purpose of data presentation.

Data were expressed as mean (standard deviation) where appropriate and groups were compared using a one-way ANOVA.

Externally administered assessments such as the Situational Judgement Tests and Prescribing Safety Assessment were not included.

## Results

We received data from all five Universities. University E was unable to provide data for the written assessments. It has a pre-clinical program only and thus was excluded from further evaluation.

### Assessment overview: objective structured clinical examinations (OSCE)

All Universities use OSCEs as part of its assessment tool. Total number of stations included in this audit was 1083. Students from Universities C and D undertake OSCEs in every year group while students from University A take them less frequently, three times each year during their period at University. Final year OSCEs comprise of two parts for Universities B and C – students who pass Part one are exempt from taking Part 2. OSCE stations ranged from 8 up to 32 per exam. There were significant differences (*p* < 0.0001) in the overall mean number of OSCE stations used at the Universities over the 5-year period (Table 1). However, this difference is lost in the most recent set of examinations (2019, *p* = 0.052). Details on the evolution of OSCE stations per examination for each University can be found in Supplementary Tables 1 and 2.

**Table 1.** Total Number of OSCE stations over 5 year period for each University

University	Total Number of OSCEs included	Total Number of Stations	Mean Stations per OSCE (SD)
A	15	210	14.0 (5.7)
B	25	216	8.5 (2.0)
C	27	317	11.7 (2.4)
D	25	340	13.6 (2.0)

*f*-ratio = 15.81; *p* < 0.0001).

### Assessment overview: written

Students from Universities A and B take at least one written assessment in each year of Medical School, while Universities C and D students sit their final written assessment in their fourth (penultimate) year. Written assessments consist of Single Best Answer Multiple Choice Question (SBA MCQ) for Universities B and D. Written papers for Universities A and C take a variety of formats. Both predominantly use SBA MCQ and Short Answer Question (SAQ), with University A also using Modified Essay Questions (MEQ).

In total, 10,534 MCQs, 134 MEQs and 302 SAQ were included in this study spanning four Universities over 3 years. There are highly significant differences in the number of SBA MCQs set between the Universities over the 3 years (*p* < 0.0001, Table 2). The summary of findings is demonstrated in Table 3 and raw data for each University examination is included in Supplementary Table 3.

**Table 2.** Radiological images in OSCEs over 5 years for each University

University	Total OSCEs	No. of Images	Mean Images per OSCE (SD)
A	15	12	0.8 (1.3)
B	25	0	0 (0)
C	27	29	1.1 (1.7)
D	25	13	0.5 (1.0)

*f*-ratio = 3.64; *p* = 0.016).

**Table 3.** Written Papers over 3 years for each University

University	Total Written Assessments	No. of SBA MCQ	No. of MEQ/SAQ/Short notes
A	15	1310	134 (MEQ) 11 (SAQ)
B	21	2720	
C	15	2310	291 (SAQ)
D	12	4194	

*f-*ratio = 237.89; *p* < 0.0001 for SBA MCQ).

### Radiological images in OSCEs

University B never used radiological images in their OSCES. Radiological images were used in OSCEs for the remaining three medical schools, with the most frequent use in University C (Table 2). Apart from University D, images were not used in assessments for MB1 and 2 OSCEs.

### Radiological images in written assessments

All four Universities include radiological images in their written assessment. Total number of images included in the assessments ranged from a minimum of 2 to a maximum of 65 during the 3-year period studied. (Table 4). Only two images were ever used (for MB 1) in University C. In all other Universities, radiological images were used throughout MB one to MB 5/6 in written assessments.

**Table 4.** Radiological images in Written Assessments over 3 years for each University

University	Total Written Assessments	No. of Images used	Mean Images per Assessment (SD)
A	15	56	3.7 (2.5)
B	21	65	3.1 (2.6)
C	15	2	0.1 (0.5)
D	12	29	2.4 (2.6)

*f*-ratio = 7.45; *p* = 0.0002).

There was a significant difference in the number of images used in both OSCE and written assessments (Tables 2 and 4). However overall, no statistical significance is seen between Universities in the number of images used in assessments when both written and OSCE assessments are combined (*p* = 0.585).

Limited data obtained for University E can be found in Supplementary Table 4.

## Discussion

Our study demonstrates wide variation in the number, types and timing of assessments deployed to evaluate medical students across Scottish Universities. There were highly significant differences in the number of OSCE stations used and the number of MCQs set over the period of study. This is in keeping with the findings of previous UK studies.^5^ While no single “best” validated method of assessing medical students exists, both MCQs and OSCEs remain popular in assessing knowledge and aspects of clinical competency, respectively.^6^ Our findings are in line with McCrorie et al’s study on the variations in medical school graduating assessments, where they argued for a standardised national medical licensing examination.^7^While providing some insight into such variations, their findings lacked the detailed analysis undertaken by the current study, in particular, its evaluation specifically targeted graduating medical students’ assessments and at one time point only.

Some medical educationalists are concerned that assessments drive learning too narrowly towards the licensing test at the expense of commitment to education and lifelong learning.^8^ There is little doubt that OSCEs, particularly with low number of stations, promote this narrow focus of learning among medical students. Many of the stations are predictable and ironically despite what is intended, a poor reflection of real-life complex scenarios. Nonetheless, the presence of radiological images in assessments indicates acceptance that radiology is an important component of medical student education.

Radiological images were used to examine medical students in all year groups in every Scottish Medical School, pointing to the importance of radiology to be so included in assessments. There is generally a good spread of images across exams and years. University B excepted, most Scottish Medical Schools utilise radiological images widely in OSCEs. It is easy to see why radiological images are easily accessible and readily used to construct around a clinical scenario to simultaneously test not just the clinical skill of reading the radiological examination, it also lends itself to assessing clinical reasoning. Educationalists from Harvard medical school found radiology OSCEs to be useful to uncover deﬁcits in individuals and groups beyond the ones detected with traditional clerkship/clinical end of block evaluations to guide remediation.^9^ A potential drawback of using image interpretation is the potential for assessment content to be leaked between cohorts, therefore its use in clinical reasoning scenario may be more appropriate.

Our data show that Scottish medical students encounter radiological images infrequently in both written and OSCE examinations. This ranged between an average of around 2.4 images per year for written assessments and 0.6 for OSCE examinations. Of note, however, radiological images are included in every written assessment in every year. This is an interesting finding which raises some important questions. Are students adequately prepared and taught radiology? Who is doing the teaching? Studies have shown a large variation in radiology teaching in medical schools – despite published curriculum recommendation.^10^ Allocated time for radiology teaching in undergraduate medical schools Scotland is low, and apparently decreasing, when compared to other European countries.^11,12^ In fact a recent national analysis of medical school timetable made no mention of radiology at all.^13^ Surveys demonstrate medical students feel poorly prepared for clinical practice with regard to radiological examinations and clinical leaders express a need for more radiology input into medical student education to prepare them for clinical practice.^14,15^ There is a persistent trend of non-radiologists teaching radiology.^16^ Radiology in the 21st century is a complex, expensive and vital tool in modern medicine, and the appropriateness of non-radiologists teaching the subject warrants further discussion.

The GMC plans to introduce a Medical Licensing Assessment to demonstrate those who obtain medical registration with a license to practice medicine in the UK can meet a common threshold for safe practice. It will consist of two parts: Applied Clinical Knowledge Test, run by the GMC, will be an online SBA MCQ assessment; while Clinical and Professional Skills assessments will be conducted by individual medical schools with GMC set requirements.^17^ A content map of presentations and conditions have been clearly laid out.^18^ In the Clinical Imaging section, two presentations and 30 conditions are listed. Many of those included conditions are complex and will require significant radiology input to deliver teaching of this extensive content.

A potential limitation of our study is the inclusion of a small number of Universities. However, the included Universities represent all medical schools providing both pre-clinical as well as clinical medical education for Scotland and is a comparable number when compared to other countries with similar sized population (for example : Singapore and Slovakia have 3, Norway and Denmark have four and Finland has five medical schools). It is further mitigated by one of the strengths of this study: the detailed evaluation of assessments across each University over a period of time. Another potential limitation may be the lack of detailed information on what radiological images were used in assessments. The potential to disrupt future assessments was felt to outweigh any minor interest this information may provide and was deliberately excluded from the date collection process.

## Conclusion

This is the first study to evaluate and quantify the use of radiological images in medical student assessments. In this detailed longitudinal study, we have shown that there are significant differences in medical student assessment across Scottish Universities. Given the importance of Radiology in modern medicine, the frequency and differences in which radiological images were used in assessments across Universities should be addressed.
